# Status non‐epilepticus

**DOI:** 10.1002/epd2.70051

**Published:** 2025-06-04

**Authors:** Ronen Spierer, Moshe Herskovitz

**Affiliations:** ^1^ Rappaport Faculty of Medicine Technion – Israel Institute of Technology Haifa Israel; ^2^ Department of Neurology Rambam Health Care Campus Haifa Israel

**Keywords:** functional status, misdiagnosis, psychogenic non‐epileptic seizures (PNES), status epilepticus, status non‐epilepticus

## Abstract

**Objective:**

Status non‐epilepticus is characterized by recurrent or prolonged psychogenic non‐epileptic seizures (PNES), which are often mistaken for status epilepticus (SE). This study focuses on the misdiagnosis of convulsive SE in patients whose seizures were of psychogenic origin.

**Methods:**

We analyzed 13 events of 8 patients initially misdiagnosed as having convulsive SE. The diagnostic evaluation included a review of their clinical presentation, treatment, and treatment response.

**Results:**

The patients in this study were predominantly treated with benzodiazepines (BZD). However, this treatment, together with anti‐seizure medications (ASM) was mostly ineffective, leading to tracheal intubation in eight of the cases.

**Significance:**

When a convulsive event does not cease with BZD and ASM, we suggest considering a differential diagnosis of PNES. The poor response to BZD raises several hypotheses regarding the origin of PNES.

**Key points**

- Status non‐epilepticus is defined as a recurrent or prolonged PNES.
- These events may frequently resemble status epilepticus.
- Tracheal intubation is a routinely employed intervention in managing it.
- We identified a lack of response to benzodiazepines in such events.
- Our findings offer insight into the possible origins of PNES.

## 1 INTRODUCTION

Convulsive status epilepticus (SE) is a critical medical condition characterized by recurrent or extended seizures, potentially leading to neuronal demise and multiple organ failure. Given the potential risks of SE, medical professionals consider this condition a matter of utmost concern and therefore strive to terminate the seizure expeditiously to prevent further harm to the patient. Prolonged or recurrent psychogenic non‐epileptic seizures (PNES) account for about 8% of all status episodes.^1^ These events, which were named functional status and pseudostatus, are often misdiagnosed as SE and therefore might be accompanied by iatrogenic complications. In these events, endotracheal intubation is not rare^2^ and even one case of iatrogenic death was reported.^3^

Since a single case report by David and Bone from 1985, which they named “hysterical paralysis”,^4^ case reports and several studies were published on status non‐epilepticus (SNE). This report from 1985 was not the first to describe prolonged PNES, but it was the starting point for literature inclusion in a recent review by Eilon et al., which counted a total of 260 cases.^5^ From this review, it could be concluded that this phenomenon of prolonged seizure was associated with female sex, young age, psychiatric history, low Glasgow Coma Scale (GCS), family history of epilepsy, and a recent trigger. The authors also mentioned that while video‐EEG is the gold standard for diagnosing PNES, a significant part of the studies did not use this tool. Obviously, video‐EEG is inaccessible in emergency settings, and in addition, in most cases, it is not needed, as semiological analysis alone can be quite accurate in differentiating between ES and PNES.^6^

Few statistical studies presented interesting characteristics of SNE patients. In a study conducted by Reuber et al., PNES patients who had recurrent statuses suffered from PNES from a younger age compared to PNES patients who did not present with status events. They, however, did not have a longer duration of disease.^7^ Dworetzky et al., who performed a similar comparison, found that SNE patients were treated with fewer anti‐seizure medications (ASM), were more likely to be admitted emergently to the monitoring unit, had shorter long‐term monitoring stays, and tended to be diagnosed sooner after initial presentation.^8^

As PNES are mostly short and self‐terminating events,^9^ they are usually not treated with medications and procedures. Therefore, the examination of SNE holds significance as it can offer valuable insights into PNES. For example, what PNES looks like, how ambulance paramedics and emergency department workers treat these seizures, and most interestingly, how the seizures respond to these treatments.

We have observed patients who experienced prolonged or repetitive psychogenic motoric attacks and have suggested the term *status non‐epilepticus* (“status epilepticus” + “non‐epileptic seizure”) to describe this phenomenon. With the selection of this particular term, which we believe stands out as the most suitable option for the purpose at hand, we emphasize the visual resemblance it shares with SE.

The primary aim of this study was to understand the extent of response to treatment lines and how many treatment lines were required to terminate the status event.

## 2 METHODS

We retrospectively reviewed cases of SNE that occurred at the Rambam Health Care Center between January 2013 and December 2022. All diagnoses of PNES were made by a specialized epileptologist. Because the current definition of SE is of seizure(s) lasting more than 5 min,^10^ and because the medical staff treated the cases as for SE, we included the cases of PNES that exhibited a duration exceeding 5 min or those that exhibited a rapid recurrence pattern within a time frame of greater than 5 min. Another inclusion criterion was the diagnosis of PNES (without a comorbidity of epilepsy) using a long‐term video‐EEG monitoring consecutive to the SNE episode.

The following data were collected from the medical records: age, sex, previous diagnosis of epilepsy, therapeutic status, duration and location of hospitalization, treatment, and procedures. If the patient had more than one record of SNE, we used the first for the calculation of averages and for comparison between patients.

The semiology of the SNE episodes was characterized according to Seneviratne et al.'s classification.^11^ The therapy lines were characterized based on the American Epilepsy Society guideline and treatment algorithm for convulsive SE: Stabilization phase (0–5 min of seizure activity) which includes first aid for seizures and initial assessments and monitoring; first‐line therapy (5–20 min) which includes intravenous BZD injection; second‐line therapy (20–40 min) which includes intravenous ASM load; third‐line therapy (40–60 min) which includes intravenous BZD, propofol, or barbiturates injection.^12^ We classified cases that involved intubation as the third‐line therapy.

The study design complied with all local laws and was performed in accordance with the Declaration of Helsinki. The study was approved by the Rambam Health Care Campus Institutional Review Board.

## 3 RESULTS

Thirteen cases of SNE in eight patients were included in the study. All patients' characteristics and PNES details are summarized in Table 1. Five were females (62.5%) and the average age at convulsion was 31.5 ± 8.9.

**TABLE 1:** Description of the 13 non‐epileptic status events.

Patient (m/f)		Age	False epilepsy title? (y/n)	Time from beginning of seizures to status event	Comorbidity	Location	Trigger	Last line of therapy
I (f)		28 years	Y	16 years	Pregnancy	ED	ASM discontinuation	Third
II (m)	A	37 years	Y	N/A	Nephrostomy	Hospital ward	Nephrectomy surgery	Third
	B	38 years	Y	N/A	Nephrostomy	Hospital ward	Hospitalization, fever, infection	Third
	C	40 years	Y	N/A	Nephrostomy	Home	Fever	Third
III (m)	A	45 years	N	0 year	Acute meningitis	ED	Meningitis	First
	B	48 years	Y	3 years		Home	Quarrel	First
	C	50 years	Y	5 years		Home	N/A	Second
IV (f)		32 years	Y	N/A	Pregnancy	Hospital ward	ASM discontinuation	First
V (f)		27 years	N	12 years	Pregnancy, fibromyalgia	Hospital ward	N/A	First
VI (f)	A	20 years	Y	N/A	Schizophrenia, intellectual disability	ED	N/A	Third
	B	26 years	Y	N/A	Schizophrenia, intellectual disability	Hospital ward	Acute appendicitis, hospitalization, surgery	Third
VII (m)		22 years	Y	N/A	Schizophrenia	ED	N/A	Third
VIII (f)		41 years	N	N/A	Paraplegia after surgery, CRPS	Hospital ward	Hospitalization	Third

*Note*: The table describes the patients' characteristics, as well as the contexts of the status episodes. The right row describes in what stage the convulsion stopped: initial for benzodiazepines, second for anti‐seizure medication, and third for intubation. In most cases, the last line was third. In addition, most patients had a previous misdiagnosis of epilepsy.

Abbreviations: ASM, anti‐seizure medication; CRPS, complex regional pain syndrome; ED, emergency department.

In most of the patients, a misdiagnosis of epilepsy was made prior to the event. Notably, patient I was diagnosed with epilepsy 16 years before the SNE event, and patient V had uncharacterized events for 12 years before being diagnosed with PNES. Patient III was diagnosed with PNES after his first event. However, he began an anti‐seizure treatment between his first and second events. This treatment was not discontinued even after the second event.

The PNES were mostly associated with a specific trigger. The nature of these seizures was mostly uniform, that is, a hyperkinetic seizure, with the exception of patient I, who presented with repetitive focal spasms, and patient IV, who had an exceptional convulsion. In all cases, a treatment of benzodiazepines (BZD) was used; however, with little or no improvement. Four events were terminated with the first‐line therapy and one with the second‐line therapy. The remaining eight events ended only after endotracheal intubation and injection of muscle relaxants. In six cases, the SNE led to ICU admission.

## 4 DISCUSSION

This study focused on the aspects of misdiagnosis of SNE and treatments that the patients received. Their condition was suspected to be an epileptic seizure and was usually treated with midazolam. As the response to midazolam was limited, most of the patients underwent general anesthesia. Only then, and probably due to the use of muscle relaxants or the induction of sedation, did the convulsions cease.

The rates of BZD and ASM use were higher in our study in comparison to Mezouar et al.'s study. This comparison might be surprising in light of the fact that they included only the severe SNE cases that ended up with ICU admission.^13^ The rate of tracheal intubation in our study was also higher than reported in that study and in others. While the previous studies reported a frequency that varied between 2% and 41%, in our study, 61.5% of the episodes resulted in intubation.^1^, ^2^, ^13^, ^14^, ^15^, ^16^, ^17^ An obvious explanation for this high rate is the inclusion of only status cases in our study. However, it could also be explained by the risk factors for intubation found by Viarasilpa et al., which included low GCS, receiving ASMs, and high doses of BZD administration.^2^ Indeed, the patients in our study had been matching these risk factors.

A prominent finding of this work was the high number of pregnant women among the included patients. This finding is, however, not very surprising because SNE occurs predominantly in women of childbearing age, as was mentioned by Peters et al.^18^ This finding also allows us to emphasize that overtreating pregnant women with PNES should be avoided as there might be a risk for the fetus.

The patients included in the current study were mostly treated with midazolam, and, as it was not of any help, with an ASM load. Thus, most of the convulsions were not seized after the first‐line treatment, in contrast to SE, which usually ceases after such treatment.^19^, ^20^

While we would not have expected ASM loading to be of any help, it seemed that midazolam was also relatively ineffective in managing SNE, and therefore, most of the patients were intubated. At this point, it should be asked, why did the patients continue to convulse even after receiving repeated boluses of midazolam, regarding PNES being associated with acute stress?

In order to answer this question, we should first discuss the mechanism by which midazolam, along with other BZD, affects the central nervous system. In a positron emission tomographic study by Veselis et al., it was concluded that midazolam‐induced sedation mainly involves cortical and thalamic regions.^21^ In addition, BZD receptors were found spread in low density in the brainstem.^22^

Given the strong association between PNES and post‐traumatic stress disorder (PTSD),^23^ the patients in our study probably exhibit similar cerebral alterations characteristic of those diagnosed with PTSD. These changes include amygdalar dysfunction and downregulation of GABA receptors.^24^ There is evidence that during a stressogenic process, there is an overproduction of neurosteroids, which also contributes to the downregulation of GABA receptors.^25^ The amygdala's initiation of the fight‐or‐flight response (“amygdala hijack”) maintains a connection to the brainstem, which is relatively resistant to anesthetic agents.^22^, ^26^ This explanation reinforces our previous hypothesis of PNES as a freeze‐discharge response,^27^ supported by findings of amygdala hyperactivity in association with PNES in other researches.^28^

Nonetheless, it is important to acknowledge that this inference remains speculative, as our study was observational and included only a small number of cases. An alternative hypothesis, paradoxical reaction to BZD, must also be considered. Although rare, paradoxical reactions are associated with psychiatric comorbidities.^29^ Given that psychogenic seizures have a dissociative component, the dissociative and disinhibiting effects of BZD may be responsible for the unfavorable effect in SNE, as was previously suggested.^17^

In addition, high rates of BZD prescription for PNES patients (possibly reflecting tolerance)^30^ and the general ineffectiveness of BZD in treating PTSD^31^ may each contribute to the lack of responsiveness observed in our study. Notably, SNE episodes ceased when the patients became fully asleep or anesthetized, allowing us to conclude that PNES events typically do not occur during states of deep sleep. Based on our experience, PNES do not arise during sleep, and those that appear to do so are usually preceded by an awakening just before the event.^27^

This study has few limitations as it is retrospective in nature and includes a small number of patients. In addition, we presented only cases of motoric attacks, while 11.2–42.5% of PNES are dialeptic in nature.^32^

## 5 CONCLUSION

Healthcare workers often face challenges in accurately distinguishing between SE and SNE, which frequently results in SNE cases being treated with advanced‐line therapies. To mitigate this, we recommend that, particularly when caregivers lack expertise in seizure classification, seizures be recorded whenever feasible before making treatment decisions. Involving specialized professionals, such as neurologists or epileptologists, at critical decision‐making points can further enhance diagnostic accuracy. Additionally, if first‐ and second‐line treatments for status epilepticus fail to produce the expected response, the medical team should consider the possibility of PNES.

## FUNDING INFORMATION

This research did not receive any specific grant from funding agencies in the public, commercial, or not‐for‐profit sectors.

## CONFLICT OF INTEREST STATEMENT

The authors declare that they have no known competing financial interests or personal relationships that could have appeared to influence the work reported in this paper.

## PATIENT CONSENT STATEMENT

The institutional review board waived patient consent due to the study's retrospective nature.

**Test Yourself**

1. What percentage of all status episodes are accounted for by prolonged or recurrent psychogenic non‐epileptic seizures?
  - 1%
  - 5%
  - 8%
  - 15%
  - 20%
2. All the following are associated with status non‐epilepticus, except for:
  - Female sex
  - Psychiatric history
  - Family history of epilepsy
  - A Glasgow Coma Scale of 15/15
  - Recent trigger
3. Psychogenic non‐epileptic seizures…
  - Can usually be stopped by intravenous midazolam
  - Are easily mistaken for status epilepticus
  - Are more common in men
  - Have a well‐established known pathophysiology
  - None of the above

*Answers may be found in the* *Supporting information*.

## Supporting information


Data S1.

## Data Availability

The data that support the findings of this study are available on request from the corresponding author. The data are not publicly available due to privacy or ethical restrictions.
