# Supplementary material for: Status non‐epilepticus
Source: Epileptic Disord. 2025 Jun 4;27(4):642–7. doi: 10.1002/epd2.70051 (PMC12398196; doi:10.1002/epd2.70051)
Supplement: Supplementary file 1 — Data S1. [file EPD2-27-642-s001.docx]

**Question 1:**

**Answer:** C. 8%

**Question 2:**

**Answer:** D. A Glasgow Coma Scale of 15/15

**Question 3:**

**Answer:** B. Are easily mistaken for status epilepticus
